# Supplementary figures and images for: Regulation of endoplasmic reticulum stress on autophagy and apoptosis of nucleus pulposus cells in intervertebral disc degeneration and its related mechanisms
Source: PeerJ. 2024 Apr 22;12:e17212. doi: 10.7717/peerj.17212 (PMC11044878; doi:10.7717/peerj.17212)

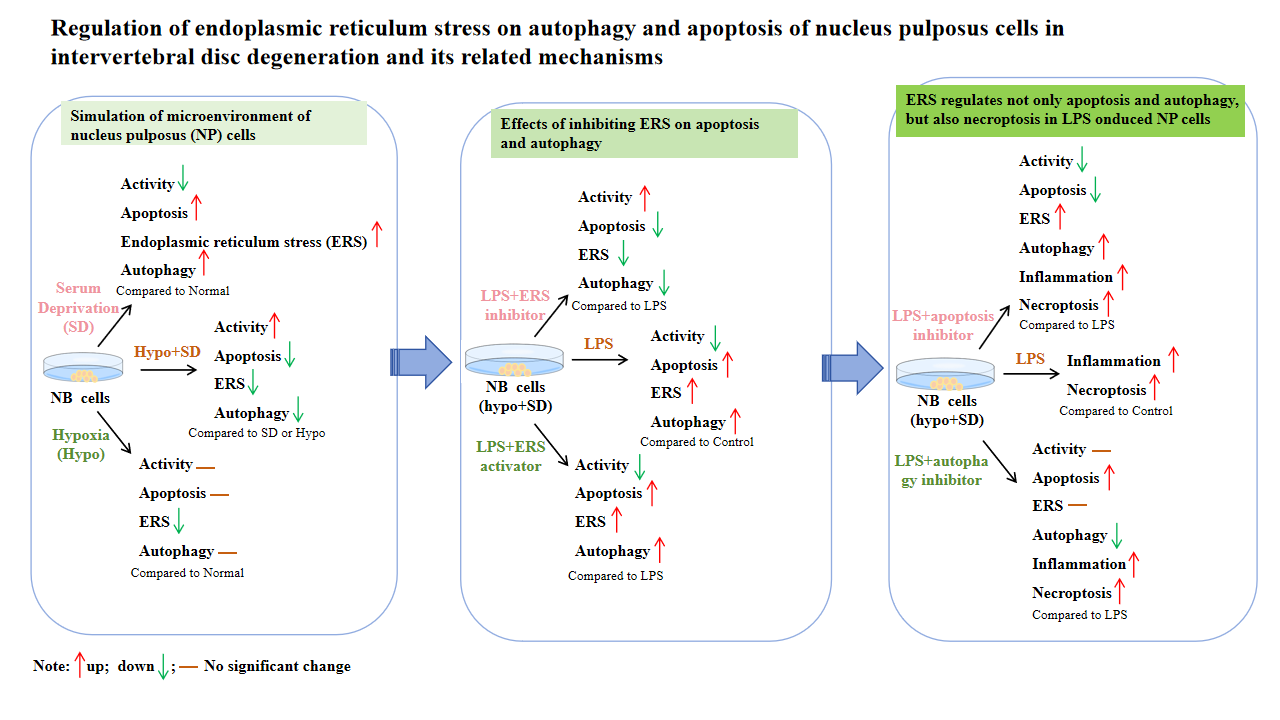

Supplement: Supplemental Information 3 [file peerj-12-17212-s003.tif]
